# In this issue of *Current Oncology*

**Published:** 2008-04

**Authors:** M. McLean

This new issue of *Current Oncology* sees a significant change of direction for the journal—for want of a better word, publication in a “hybrid format.” Although all manuscripts will continue to be published online at www.current-oncology.com as usual, not all will be published in their full form in the hardcopy issue. Papers selected for online publication exclusively will also be presented in an extended (single-page) short version in the print publication. The present hardcopy issue therefore includes two short-form manuscripts with universal resource locaters (URLs) that link to the respective complete online versions.

The hardcopy version of *Current Oncology* will carry full versions of invited editorials, unsolicited manuscripts with a broad-based appeal to readership, and selected sponsored items such as the new “info editorial” series. Conversely, manuscripts for a more specialized audience and those that need exceptional length to demonstrate their theses, may appear in full online only. The cost of publishing colour online is trivial as compared with colour in hard copy, and one interesting (and to date unexplored) asset for online publication is the ability to include selected audio and video formats such as MP3. Although such formats are perhaps more appealing to the various journals of ornithology, they may represent added value for some submissions arriving here!

The publishing world has changed dramatically in the past couple of years—a notable development being the open-access journals (of which *Current Oncology* is an example), which most major medical publishing companies such as MultiMed are now incorporating. Electronic open-access journals provide speedier turnaround from submission to publication, allow for more interactivity between authors and readers, and comply with the growing demands placed on libraries and other depositories to sustain a wide range of journals in their inventories despite limited space and tight budgets. A hybrid model can only enhance the ability of *Current Oncology* to maintain the broadest exposure within its operating budget.

It should be noted that all published manuscripts, be they online only or print, are fully indexed in our associated databases, including PubMed, Excerpta Medica, and (most recently) Index Copernicus.

